# Experimental study on fracture and fragmentation characteristics of laminated rocks under impact loading

**DOI:** 10.1038/s41598-025-33865-z

**Published:** 2026-01-12

**Authors:** Ding Deng, Yuling Li, Lianjun Guo, Gaofeng Liu, Jiawei Hua

**Affiliations:** 1 School of Materials Science and Engineering, Shenyang University of Technology, Shenyang, 110870 Liaoning China; 2 School of Architecture and Civil Engineering, Shenyang University of Technology, Shenyang, 110870 Liaoning China

**Keywords:** Energy-time density, Impact load, Acoustic emission technique, Fractal dimension, Broken and fractured rocks, Engineering, Materials science, Natural hazards, Solid Earth sciences

## Abstract

Laminated rock bodies are prevalent in engineering applications, making the investigation of their damage characteristics and evolution patterns under impact loading critical for ensuring engineering safety. Despite this importance, the relationship between energy dissipation and the crushing mode of laminated rock bodies remains underexplored, particularly regarding the influence of lamination orientation on their dynamic response. To bridge this scientific gap, this study conducts rock impact tests in the presence of various lamination angles (0°, 30°, 60°, and 90°) and employs digital image correlation (DIC) technology alongside acoustic emission (AE) monitoring and fracture sieve analysis to systematically examine how lamination orientation affects energy dissipation patterns, crack propagation behavior, and fragmentation characteristics in rocks. The results indicate that the lamination angle has a significant influence on the energy dissipation characteristics of rocks. When the lamination orientation is parallel or perpendicular to the direction of shock wave propagation, the rocks exhibit a low intensity of energy dissipation, a singular crushing mode, restricted crack propagation, and a relatively stable fragmentation process. Conversely, laminated rocks with oblique intersections demonstrate complex damage modes due to the combined effects of shear and tension, wherein significant large-scale crack propagation occurs, exacerbating structural instability. Analysis of AE *b*-values corroborates these observations, revealing that the development of microcracks in rocks with diagonally interbedded laminations along the impact direction is more prone to result in large-scale destabilization and damage. This study elucidates the damage mechanisms and directional characteristics of laminated rocks subjected to impact loading, thereby providing a vital theoretical foundation and technical support for the safe design of engineering structures and related construction practices.

## Introduction

Laminated rock bodies are extensively utilized in mining, tunneling, underground engineering, and national defense sectors. The fracture and crushing characteristics of these materials under impact loading significantly influence the stability and safety of engineering structures. However, the complex anisotropic nature of laminated rock masses makes it challenging to accurately predict and control their damage patterns, particularly under dynamic impact loading conditions, where the mechanisms of crack propagation and energy dissipation remain incompletely understood.

Currently, both domestic and international scholars have conducted numerous investigations to explore the mechanical properties of rocks subjected to static or quasi-static loads. Berthaud^1^ analyzed the degradation behavior of brittle rock materials during quasi-static loading, highlighting the impact of localization phenomena on the stress-strain relationship of rocks. Zou et al.^2^ examined the mechanical behavior of rock-like brittle materials, such as gypsum, under dynamic and quasi-static loading conditions, revealing that shear damage predominantly occurs under dynamic impact loading, while tensile fractures prevail under quasi-static loading. Li et al.^3^ examined the mechanical properties of rocks under coupled static-dynamic loading with enhanced experimental setups, discovering that the strength of rocks exceeded that observed under either static or dynamic loading alone, provided the axial prestress remained below the elastic limit, and that it increased with higher strain rates. Feng et al.^4^ explored the mechanical behavior of rocks under combined static-dynamic loading, demonstrating that the initial static load significantly impacted total energy dissipation and rock response, with a notable tendency to soften as the initial static load increased.

In recent years, attention has turned to the dynamic response characteristics of laminated rocks under impact loading. Liu et al.^5^ examined the energy dissipation characteristics of horizontally laminated composite rocks and found that as the impact rate increased, the damage behaviors of various incidence states tended to converge, with energy dissipation being quadratically related to the fractal dimensions. Hao et al.^6^ examined the mechanical properties of laminated sandstone under varying fracture modes, establishing that the angle of lamination significantly influenced crack propagation paths and fracture toughness, with the maximum tensile stress criterion providing a more accurate prediction of the fracture behavior in laminated sandstone. Zhao et al.^7^ investigated the fracture behavior of prefabricated cracked sandstone using SHPB experiments and observed the coexistence of shear and tensile cracks, with shear damage being more pronounced; furthermore, they noted that higher impact rates could substantially enhance the fracture strength of the rocks. Additionally, Xu et al.^8^ explored the creep structural effects of laminated rock bodies and found that laminated structure significantly influenced creep parameters, leading to accelerated creep at high stress levels and a sharp release of AE energy prior to damage.

To further examine the fracture-shattering mechanisms of laminated rocks under impact loading, researchers have employed digital image correlation (DIC) techniques in conjunction with acoustic emission (AE) monitoring to quantitatively analyze the characteristics of crack propagation and damage evolution. Li et al.^9^ investigated the anisotropy and AE energy evolution of laminated sandstones subjected to uniaxial compression, revealing that the presence of laminated surfaces significantly enhances the rock’s anisotropy. They also noted that the decreasing trend of the b-value could serve as an early warning indicator of rock instability. Song et al.^10^ explored the AE source characteristics of granite under uniaxial compression and found a positive correlation among the absolute AE energy, cumulative counts, and peak intensity of the rock. Gautam and Gutierrez^11^ examined the AE characteristics of synthetic rocks, discovering that AE frequency information could be utilized to identify the formation and propagation of new cracks, while the rock’s brittleness influences AE cumulative energy and frequency distribution. Ma et al.^12^ studied the AE characteristics of laminated concrete blocks during static rupture and identified distinct distribution characteristics of AE events at various load stages. Qi et al.^13^ investigated the mechanical damage characteristics of laminated composite rocks using AE and DIC techniques, notably constructing a damage ontology model. Xiong et al.^14^ performed a quantitative analysis of the crack propagation process of laminated rocks under uniaxial compression through DIC and AE techniques, introducing a covariance matrix-based method for crack type identification. Fan et al.^15^ studied the dynamic impact responses of laminated rock bodies, employing the DIC technique to elucidate the influence of the laminations’ angle on the region of strain concentration. Haile et al.^16^ applied the DIC technique to measure the anisotropy of shale rocks, discovering that the orientation of the laminae affects local deformation patterns and governs the failure modes of shale. In addition, the fractal theory proposed by Mandelbrot^17^ was utilized by Tyler et al.^18^ to develop models for rock fragment grain size distribution. In continuing, Chen et al.^19^ further advanced this approach by integrating fractal theory to formulate a model for the mechanical behavior and fine structure of dynamic crack propagation in rocks .

In addition to single laminated rock masses, engineering surrounding rocks commonly exhibit composite laminated structures composed of alternating weak and strong layers. Such structures not only display pronounced anisotropy but also involve coupled effects of interfacial constraints and material heterogeneity, thereby altering the proportion of crack types, post-peak instability characteristics, and energy release modes. With increasing lithological homogeneity, tensile cracks become more dominant, while crack propagation paths and geometric complexity parameters exhibit a quantifiable dependence on homogeneity. Meanwhile, different failure modes reconstruct energy transfer pathways and influence the formation and spatial distribution of critical energy release zones^20^. By introducing a homogeneity coefficient and an energy weighting factor, the mechanism of “strain localization–rapid energy release–sudden post-peak instability” is quantitatively characterized, providing important insights for establishing instability criteria of laminated and composite structures under dynamic disturbances^21^.

In summary, this paper proposes an innovative index, the so-called energy-time density, to evaluate the energy dissipation of rocks subjected to impact loading. Concurrently, it systematically examines the crack extension paths and rupture mechanisms of laminated rocks under similar conditions, utilizing dual monitoring techniques, specifically DIC and AE. The study quantifies the distribution of fracture scales through the application of fractal theory and systematically assesses the fractal characteristics of impact-crushed rocks. The primary aim is to elucidate the intrinsic relationship between structural features and dynamic fracture behavior, thereby providing valuable research insights into the fracture and fragmentation characteristics of laminated rocks under impact loading.

## Methodology

### Specimen selection and processing

The objective of this study is to examine the fracture and fragmentation characteristics of laminated rocks subjected to impact loading. Through the execution of impact dynamics tests on laminated rock specimens, we systematically captured high-speed photographic images and AE signals during the impact events to conduct a detailed analysis of dynamic fracture behavior. Additionally, we performed particle size analysis on the rock mass post-impact to uncover its fractal characteristics and energy dissipation mechanisms.

**Fig. 1:**
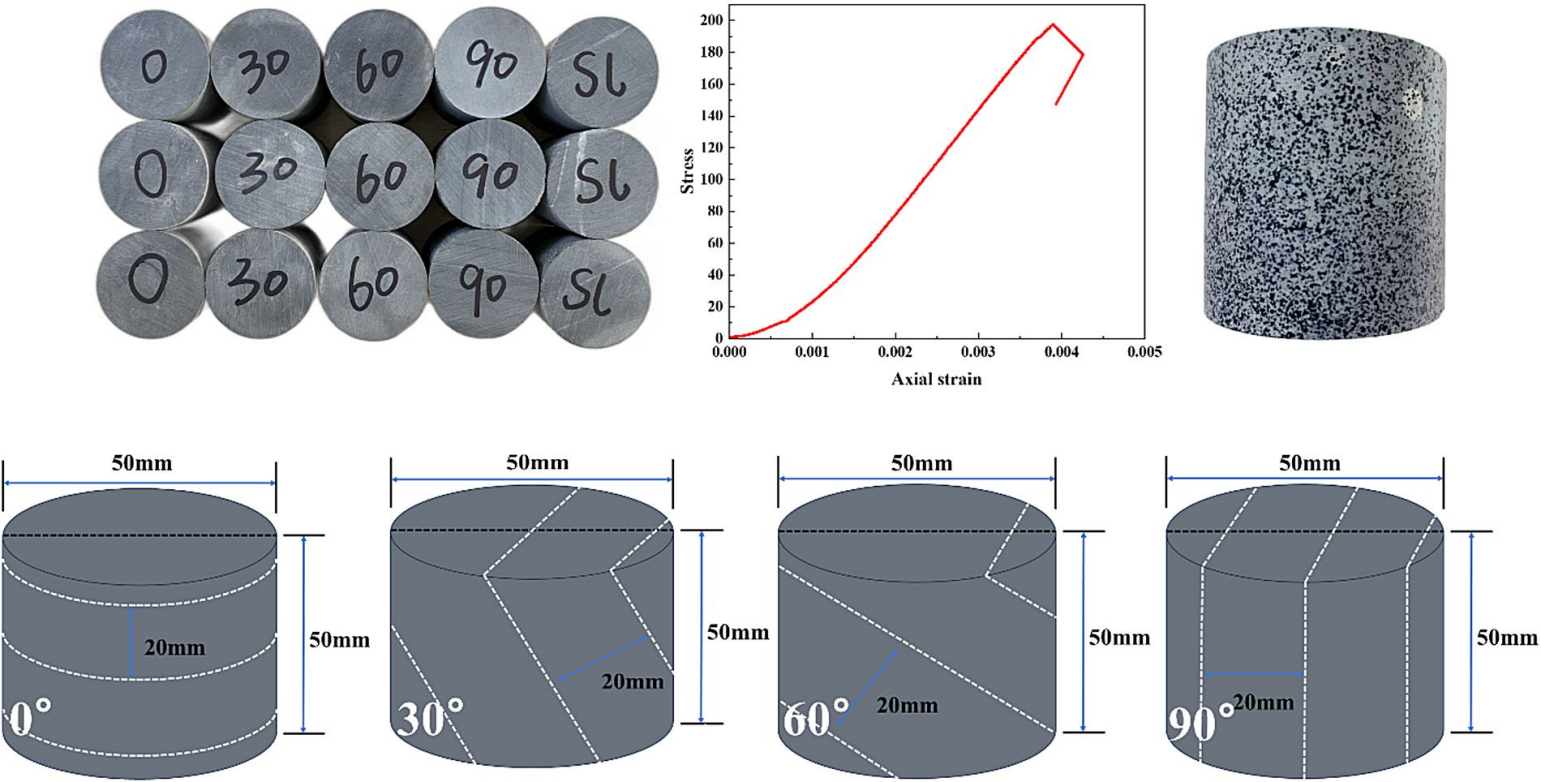
Diagram of test specimens of laminated rock.

To minimize the influence of variations in inherent initial properties, rock blocks were collected from the same location within a mine in Jiujiang, Jiangxi Province, China. In accordance with the experimental requirements, four standard cylindrical slate specimens (φ50 mm × 50 mm) were prepared. In this study, the angle between the normal to the bedding plane and the direction of impact loading is defined as the “bedding angle.” Specimens with different bedding angles were fabricated using a wet coring/sampling procedure, as shown in Fig. 1. Basic physical and mechanical tests were conducted on the specimens, and together with previous studies^22–24^, it was verified that although the bedding orientation varies slightly, the mechanical properties of the same rock type do not exhibit significant differences. The layered rock used in the experiments has a density of 2653.88 kg/m³, a uniaxial compressive strength of 197.62 MPa, and a Young’s modulus of 50.80 GPa. To better describe and analyze crack evolution in the layered rock specimens during impact tests, digital image correlation (DIC) was employed to record the cracking process. For DIC speckle preparation, the specimen surface was first cleaned to remove oil and dust; black spray paint was then applied by light tapping and/or misting from a distance to form random speckles after complete drying, as illustrated in the figure.

### Test setup and methods

**Fig. 2:**
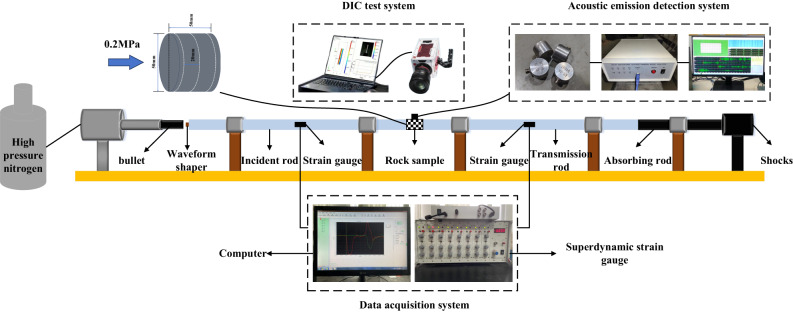
Schematic representation of the split Hopkinson pressure bar (SHPB) experimental system.

The SHPB system was utilized in this experiment, as depicted in Fig. 2. The Hopkinson system mainly comprises impact bars, impingement bars, incidence bars, and transmission bars. These components are constructed from ultra-high-strength low-alloy steel, featuring a density of 7800 kg/m³ and a modulus of elasticity of 211 GPa. Thin copper sheets, affixed to the free end of the impingement rods, serve as impulse shapers, while a pair of strain gauges is positioned at the midpoint of both the impingement and transmission rods to capture strain signals during the dynamic loading process. The geometry of the pulse shaper consists of a copper disk measuring 10 mm in diameter and 2 mm in thickness, which was glued to the left end of the incident rod to promote the dynamic equilibrium of the specimen. During the impact tests, the impact air pressure was controlled at 0.2 MPa.

The AE system utilized in this study was developed by Beijing Soft Island Times Technology Co., Ltd., incorporating an 8-channel DS5 monitoring setup. The system operates with a maximum sampling frequency of 10 MHz; however, the experiments detailed in this paper are conducted at a sampling frequency of 3 MHz. The AE preamplifier features a fixed gain of 40 dB, while the utilized AE sensor is the RS-2 A model with an M5-KY interface. Its operational response frequency ranges from 60 to 400 kHz, centering around a frequency of 150 kHz.

Previous studies have conducted AE tests on rocks under SHPB conditions, demonstrating that AE can serve as an effective auxiliary technique for characterizing the impact-fracture process. For instance, LIU et al.^25^ explicitly proposed an AE waveform acquisition protocol in SHPB impact-loading experiments (40 dB preamplification, a relatively high sampling rate, and threshold triggering), and systematically analyzed the amplitude distribution and frequency characteristics of AE under impact loading. Therefore, considering the instrument’s synchronous sampling capability, triggering scheme and full-waveform acquisition capacity, as well as prior applications of AE methods in dynamic impact (SHPB) tests, the AE measurement chain adopted in this study is suitable for AE data acquisition and statistical analysis in rock SHPB dynamic experiments.

Although the above system is applicable to dynamic AE acquisition in SHPB tests, it also has certain limitations: under impact conditions, the dominant frequency of AE may reach 300–500 kHz. Liu et al.^25^ reported that, under SHPB impact loading, the recorded AE waveforms exhibit a dominant frequency range of 300–500 kHz, while frequency components above 500 kHz are not significant. This may introduce systematic errors into the results.

Despite these limitations, they do not affect the overall regularity of the conclusions. All experiments were completed using the same experimental setup; thus, the systematic errors can be approximately regarded as mutually offset. The conclusions of this study emphasize the relative evolutionary trends during the impact process, rather than relying on fine details of absolute spectral data. In summary, the AE data in this study can be used to reveal the evolution of rock damage under impact loading.

The DIC image acquisition system comprises an S1315M/C CMOS ultra-high-speed camera, image acquisition software, and a high-power LED light source. The full-frame resolution is 1280 px × 1024 px, and the frame rate for this study is set at 15,000 fps. Prior to testing, the surface of the specimen undergoes thorough preparation, including cleaning with alcohol, followed by the decision to apply a uniform layer of white matte primer if needed, to satisfy the requirements of the DIC test. After the white paint dried, the black scattering was sprayed to further meet the DIC testing specifications.

In this study, two-dimensional digital image correlation (DIC) analysis was performed using the Ncorr software to inversely determine displacement and strain fields from speckle image sequences on the specimen surface. The image acquired in the unloaded state of the specimen was taken as the reference image, and the region of interest (ROI) was defined on the reference configuration. The ROI covered only the actual or primary deforming region of the specimen, while avoiding background areas and locally complex deformation zones induced by end gripping or constraints, thereby reducing the influence of boundary effects on result interpretation. To ensure correlation accuracy, the entire image sequence was acquired under identical imaging conditions, and the relative position between the camera and the specimen remained fixed throughout the experiment. The ROI was specified using Set Reference ROI and interactively delineated with Draw ROI.

During DIC parameter configuration, a subset-based local correlation method was adopted as the core computational strategy, and the displacement field was solved using the iterative optimization procedure embedded in Ncorr. Considering the trade-off between local detail resolution and noise suppression, the parameter selection followed the principle that the subset radius should be as small as possible to avoid excessive smoothing, and a stable parameter combination was determined through multiple trial calculations and iterative refinement. In this study, the subset radius was set to 33 px, and the subset spacing was set to 2 px, corresponding to a computation grid step of 2 px. Subpixel displacement was obtained using the subpixel interpolation/fitting strategy provided by the software, according to the Ncorr configuration options.

Subsequently, seed points were placed within the ROI to initiate the correlation search. Seed points were preferentially selected at locations that remained within the field of view throughout the deformation process and were distributed as uniformly as possible to enhance the stability and coverage of the correlation search within the ROI.

To improve computational efficiency, OpenMP parallelization could be enabled, and the number of parallel threads was set according to the available CPU cores. If parallelization was not enabled, the computation defaulted to single-thread mode. The parallel configuration was used to accelerate the correlation calculations and iterative solution process within the ROI, thereby reducing the overall computation time.

In the result output and post-processing stage, displacement values were converted from pixel units to physical units based on calibration-derived scale factors (e.g., mm/px) using the unit conversion function under Units Options, and the displacement and strain fields were exported. For visualization, spatial distributions of displacement and strain were plotted with respect to the reference configuration (Lagrangian framework), providing an intuitive representation of specimen deformation and strain distribution.

### Stress equilibrium

**Fig. 3:**
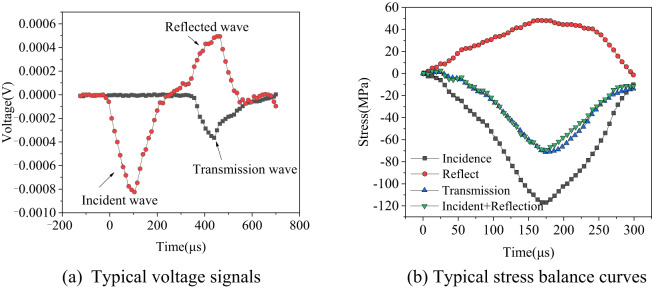
Typical stress wave signals and stress balance calibration curves. (**a**) Typical voltage signals, (**b**) Typical stress balance curves.

The attainment of a dynamic stress balance state prior to the damage of the rock specimen is fundamental in determining the validity of the SHPB test results. Following the guidance provided by ISRM, the test utilizes a purple copper sheet for waveform shaping. This approach enhances the rise time of the loading waveform, effectively filters out high-frequency components, and mitigates dispersion effects, thereby facilitating stress balance at both ends of the specimen throughout the testing process. Prior to the main test, a pre-test is performed to confirm the stability of the apparatus and to monitor the stress balance state during the testing phase. Figure 3 is a typical stress wave signal and a stress balance calibration curve. Figure 3(a) illustrates a representative stress wave pattern from the loaded rock specimen, where the incident, transmitted, and reflected waves display sinusoidal characteristics, meeting the stress balance criteria for the experiment. Figure 3(b) presents the typical stress balance test curves, with the sum of the incident and reflected wave curves closely matching the transmitted wave curves. This indicates that the testing apparatus satisfies the stress balance requirements inherent to the impact loading process.

## Results and analysis

### Characteristics of energy evolution of laminated rocks under impact loading

In Hopkinson’s test, the energy of the incident wave is transmitted to the specimen via an elastic rod. A portion of this energy is reflected and transmitted, while another part is absorbed by the rock specimen itself. The absorbed energy primarily facilitates the development of crack propagation within the rock. A minor fraction of the energy is dissipated through various modes, including acoustic, optical, thermal, radiant, and kinetic processes. In this context, the dissipated energy is often negligible. Thus, the rock specimen utilizes the absorbed energy for the deformation and damage processes associated with the expansion of cracks, as a result of energy dissipation. The quantification of this energy can be performed as follows^26,27^:

1 $${W_{\mathrm{i}}}=\frac{{{A_0}}}{{{\rho _0}{C_0}}}\int_{0}^{t} {\sigma _{{\mathrm{i}}}^{2}} (t){\mathrm{d}}t$$

2 $${W_{\mathrm{r}}}=\frac{{{A_0}}}{{{\rho _0}{C_0}}}\int_{0}^{t} {\sigma _{{\mathrm{r}}}^{2}} (t){\mathrm{d}}t$$

3 $${W_{\mathrm{t}}}=\frac{{{A_0}}}{{{\rho _0}{C_0}}}\int_{0}^{t} {\sigma _{{\mathrm{t}}}^{2}} (t){\mathrm{d}}t$$

4 $${W_{\mathrm{d}}}={W_{\mathrm{i}}} - {W_{\mathrm{r}}} - {W_{\mathrm{t}}}$$

where *W*_*i*_, *W*_*r*_, *W*_*t*_, and *W*_*d*_, are incident, reflected, transmitted, and absorbed energies, respectively; in addition, *σ*_*i*_, *σ*_*r*_, and *σ*_*t*_ in order are incident, reflected, and transmitted stresses, *A*_*0*_ is the cross-sectional area of the rod, *C*_*0*_ is the elastic wave velocity in the compression rod, and *ρ*_*0*_ is the density of the elastic rod.

To minimize the effect of specimen volume, the energy dissipated per unit volume is defined as the energy density:

5 $${U_d}=\frac{{{W_d}}}{V}$$

where *U*_*d*_ is the energy density, *W*_*d*_ is the absorbed energy, and *V* is the volume.

Most current research on rock under impact loading aims to eliminate the influence of specimen size effects on energy dissipation. Specifically, the choice of specific energy absorption is utilized to characterize the capacity of a unit volume of specimen to absorb energy. The structural characteristics of energy encompass not only the magnitude of the energy but also the temporal dynamics associated with its action. The stress wave response within the specimen over time significantly influences the energy behavior of the material. Therefore, we define this relationship through the intensity over time parameter, referred to as *E*_*VT*_. In conjunction with our previous studies, the energy–time density exhibits higher sensitivity than several other energy-based evaluation indices^28^.

The energy-time density of the rock is obtained from the stress-strain, which responds to the energy dissipated per unit volume of rock per unit time, and is calculated as follows:

6 $${E_{VT}}=\frac{{{U_d}}}{T}$$

where *E*_*VT*_ stands for the energy-time density, *U*_*d*_ is the energy consumption density, and *T* is the reflected wave action time.

**Fig. 4:**
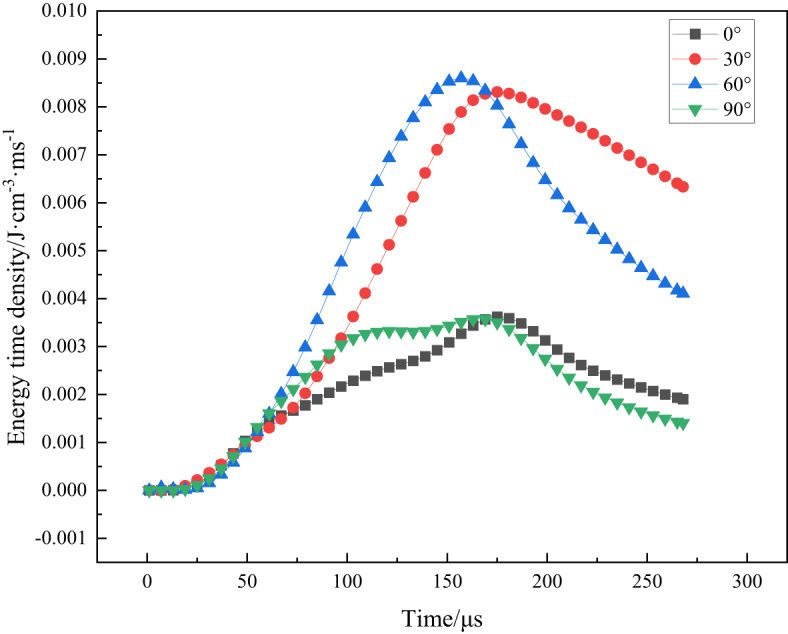
Energy-time density evolution curves for various lamination angles.

In Fig. 4, the evolution of energy-time density in rocks with varying lamination angles (0°, 30°, 60°, 90°) under the same air pressure over time is illustrated. The plotted results indicate that the lamination angle significantly influences the energy dissipation characteristics of the rocks. Specifically, the peak energy-time density varies with the layer angle, with 30° and 60° layers exhibiting larger peaks, signifying increased energy absorption and dissipation under impact loading. Conversely, the 0° and 90° layers display smaller peaks, reflecting lower energy absorption and dissipation. Notably, the peak energy-time density for the 0° and 90° laminations is reduced, indicating diminished energy dynamics. Furthermore, as the lamination angle increases, the peaks of energy-time density manifest earlier, peaking around 150 µs before gradually declining and stabilizing. The 0° and 90° layers display relatively delayed peaks with a more gradual decrease, whereas the 30° and 60° layers reach their peaks slightly earlier and exhibit a steeper decline.

When the lamination angles are set as 30° and 60°, the propagation direction of the stress wave aligns more consistently with the orientation of the lamination structure, leading to intensified deformation and destruction of the rock, resulting in greater energy dissipation and, consequently, higher energy-time density. In contrast, with lamination angles of 0° and 90°, the lamination structure impedes the propagation of the shock wave, obstructing energy transfer. This reduced transfer leads to less damage to the rock under impact and consequently lower energy dissipation, as reflected by the decreased energy-time density.

### Characteristics and mechanism of crack extension in laminated rocks under impact loading

#### DIC digital imaging technology

The DIC technique is primarily employed to monitor the progression of displacement and strain fields on the surface of specimens throughout the experimental process, thereby facilitating the assessment of the rock failure mechanisms. In the context of impact testing, the DIC technique effectively captures the strain distribution, crack propagation, and localized damage characteristics of rocks subjected to impact loading^29^.

**Fig. 5:**
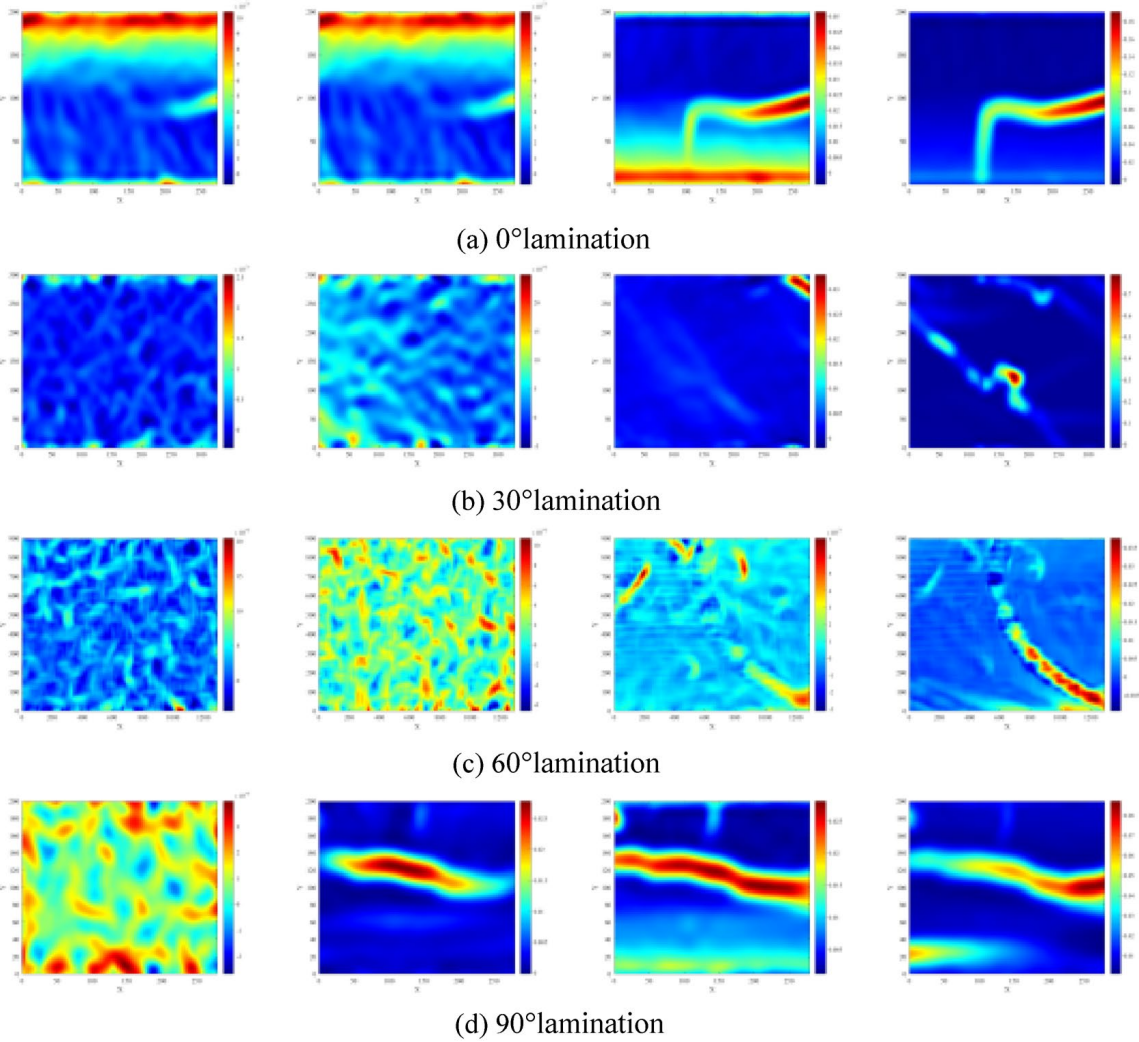
Evolution of the maximum principal strain on the surface of various laminated rock specimens. (**a**) 0° lamination, (**b**) 30° lamination, (**c**) 60° lamination, (**d**) 90° lamination.

**Fig. 6:**
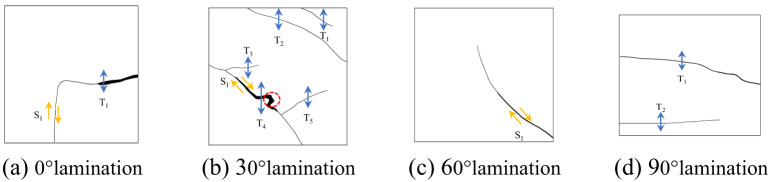
Surface cracking map of various laminated rock specimens. (**a**) 0°lamination, (**b**) 30°lamination, (**c**) 60°lamination, (**d**) 90°lamination.

Figure 5 illustrates the evolution of the fracture and stress fields on the surfaces of specimens with varying lamination angles, as assessed using the DIC technique. Additionally, Fig. 6 further presents a schematic diagram of extracted cracks based on crack identification: when the crack normal is nearly aligned with the direction of the maximum principal stress and the displacement on both sides is dominated by normal opening, the crack is classified as a tensile crack; when the crack forms a relatively large angle with the compression axis and exhibits pronounced tangential offset across the two faces, it is classified as a shear crack. Where S and T denote shear and tensile damage, respectively. Under the same impact air pressure, the DIC results, along with the crack diagrams for various lamination angles, reveal significant features and distinctions in the deformation damage patterns of laminated rocks.

The variations in rock damage modes at various lamination angles essentially stem from the orientation of the laminae in relation to the principal stress direction, which directly influences the distribution of shear and tensile stresses within the rock. As shown in Figs. 5(a) and 6(a), when the bedding angle is 0°, the laminae are oriented perpendicular to the impact direction, leading to the generation of tensile stresses along the laminae under the impact load. This configuration increases the likelihood of slip shear occurring on the weaker side of the rock laminae, resulting in the formation of penetrating tensile shear cracks. In contrast, as shown in Figs. 5(d) and 6(d), when the bedding angle is 90°, the lamination surface aligns parallel to the impact direction, where the impact load primarily induces normal tensile stresses along the lamination surface. As a result, the rock is more predisposed to experience tensile damage that is perpendicular to the lamination surface, indicating a predominance of tensile fractures. As shown in Figs. 5(b, c) and 6(b, c), for bedding inclinations of 30° and 60°, the angle between the laminae and the loading stress introduces a decomposition of the loading stress into components parallel and perpendicular to the laminae. The former component induces shear slip, while the latter causes tensile cracking. Consequently, both shear and tensile cracks manifest within the rock, resulting in a complex crack network characterized by poor damage continuity and a mixture of shear and tensile damage modes. The greater the coupling effect between shear stress and tensile stress, the more complex the crack path becomes, leading to reduced continuity in the damage.

In summary, it is evident that the laminae angle significantly influences the damage mechanism of the rock under impact loading conditions. A single damage mechanism predominates in parallel (0°) or perpendicular (90°) laminae, whereas a composite damage mode emerges from the interplay of shear and tensile stresses in diagonally intersecting laminae (30° and 60°). For 0° laminated rocks, tensile-shear composite cracks along the laminae dominate, with the weak surface of the laminae serving as a pathway for crack penetration and expansion, thereby exhibiting a distinct damage mode and a well-defined path of crack progression. In 90° laminated rocks, the tensile damage mode is pronounced, with cracks primarily oriented perpendicular to the laminae direction, resulting in clearer crack paths and relatively straightforward damage modes. In contrast, the 30° and 60° laminated rocks exhibit a mixed damage mechanism of shear and tension, with cracks propagating along and across the bedding surface, yielding a complex fracture network that diminishes damage continuity. This complex mechanism primarily arises from the synergistic effects of shear and tensile stresses, leading to increased uncertainty in the crack path.

From a fracture-mechanics perspective, the crack propagation observed by DIC can be interpreted as a mixed-mode fracture process involving Mode I (opening) and Mode II (sliding) governed by bedding-induced weak planes. When the bedding orientation is either 0° or 90° relative to the impact-induced principal-stress direction, crack growth is dominated by a single prevailing mechanism: specimens with 0° bedding are more prone to developing tensile–shear mixed cracks that propagate along or near the bedding weak plane, whereas specimens with 90° bedding preferentially form tensile cracks perpendicular to the bedding. Consequently, the macroscopic crack trajectories are clearer and the failure patterns are relatively simple. For specimens with bedding angles of 30° and 60°, decomposition of the external load on the bedding plane causes the crack tip to experience both normal opening and tangential sliding components, resulting in simultaneously significant *K*_*Ⅰ*_ and *K*_*Ⅱ*_ (mixed-mode conditions). Under such circumstances, cracks are more likely to deflect and branch, and to compete between “bedding-parallel” and “bedding-crossing” propagation paths, ultimately producing the more complex crack networks and poorer failure continuity observed in the DIC results.

#### AE RA-AF characterization

The method of classifying tensile and shear cracks in laminated rocks by the average frequency and rise angle in the AE signal has been extensively utilized for crack determination in rock materials^30,31^.

7 $$RA=\frac{{RT}}{B}$$

8 $$AF=\frac{{CR}}{{DT}}$$

where *AF* denotes the average frequency, defined as the ratio of the ringing count *CR* to the acoustic-emission signal duration *DT*. *RA* represents the rise angle, calculated as the ratio of the rise time from the threshold to the maximum amplitude *RT* to the amplitude *B*.

**Fig. 7:**
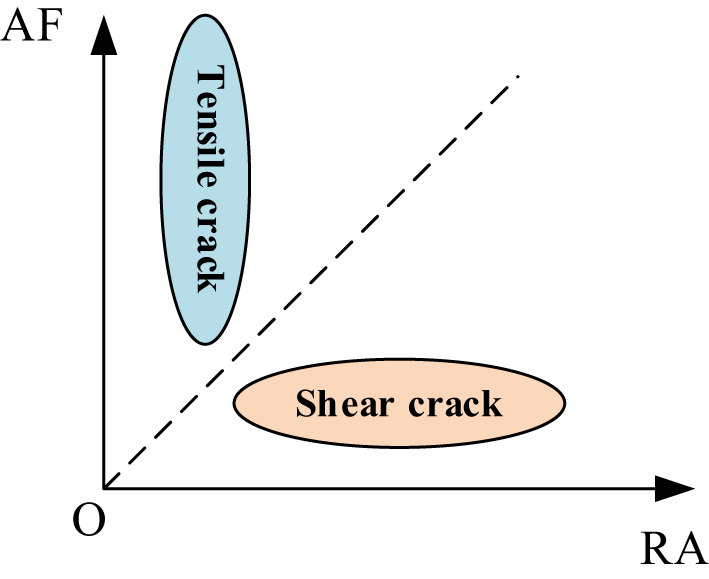
Relationships among RA-AF crack types.

The ratio of AF to RA (AF/RA) is utilized to distinguish between crack types following the normalization of both metrics; specifically, an AF/RA value greater than 1 indicates a tensile crack, while a value less than 1 denotes a shear crack^32^, as shown in Fig. 7.

The loading levels were classified according to the peak impact stress, and the maximum loading level was defined as the level corresponding to the highest peak impact stress^33^.The RA–AF criterion can be regarded as an indirect indicator of crack-propagation mode: tensile-type events typically correspond to crack growth dominated by Mode I opening, whereas shear-type events correspond to crack growth dominated by Mode II sliding. Accordingly, variations in the proportions of shear versus tensile events with bedding angle can be used to infer the evolving trend of mixed-mode contributions at the crack tip during impact, and they corroborate the competitive relationship observed in the DIC results between “bedding-parallel sliding” and “bedding-crossing tensile cracking” propagation paths.

**Fig. 8:**
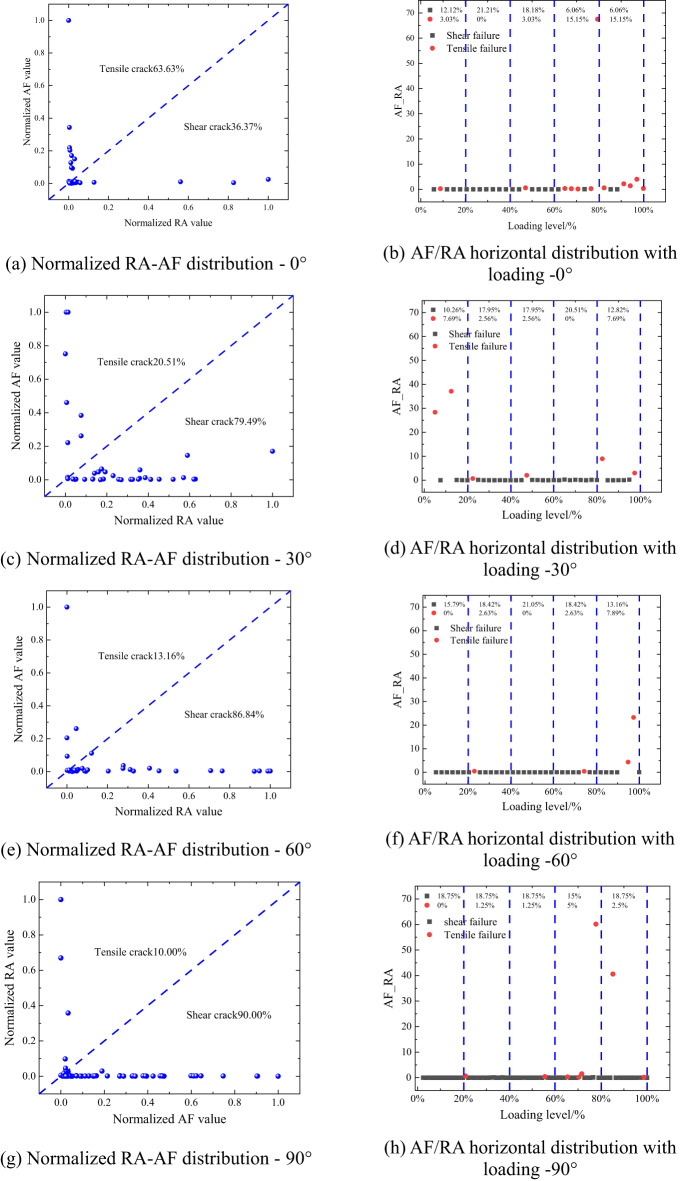
Normalized RA-AF and AF/RA distributions of laminated rocks. (**a**) Normalized RA-AF distribution − 0°, (**b**) AF/RA horizontal distribution with loading − 0°, (**c**) Normalized RA-AF distribution − 30°, (**d**) AF/RA horizontal distribution with loading − 30°, (**e**) Normalized RA-AF distribution − 60°, (**f**) AF/RA horizontal distribution with loading − 60°, (**g**) Normalized RA-AF distribution − 90°, (**h**) AF/RA horizontal distribution with loading − 90°.

Figure 8 presents the acoustic-emission characteristics of rocks with different bedding angles (0°, 30°, 60°, and 90°) as a function of loading level under the same impact-gas pressure. Panels (a), (c), (e), and (g) show the normalized RA–AF distributions, whereas panels (b), (d), (f), and (h) show the AF/RA distributions.

As shown in Fig. 8 (a), for layers oriented at 0°, the distribution indicates 36.37% shear damage and 63.63% tensile damage; as shown in Fig. 8 (c), at 30°, 79.49% shear damage and 20.51% tensile damage; as shown in Fig. 8 (e), at 60°, 86.84% shear damage and 13.16% tensile damage; and, finally, as shown in Fig. 8 (g), at 90°, 90.00% shear damage and 10.00% tensile damage. This data reveals that shear damage predominates when the lamination direction aligns with the loading direction, whereas tensile damage is more prevalent when the lamination is perpendicular to the loading direction. As the lamination angle increases, the proportion of shear damage escalates, suggesting that higher lamination angles further facilitate shear damage dominance during the rock destabilization process. The increasing angle between the loading direction and the angle of the laminae intensifies the constraint effect at the laminae interface, thereby promoting the occurrence of shear damage within the rock structure.

Examining the AF/RA distribution under varying loading levels, as shown in Fig. 8(b), (d), (f) and (h), it is observed that at low loading levels (below 20%), AE events are sparse across rocks with different laminations, signifying that elastic deformation prevails at this stage, with minimal crack initiation. In contrast, at medium loading levels (20%-60%), there is a marked increase in AE events, particularly in 0° and 30° laminated rocks, indicative of active crack propagation and a dominance of shear damage signals. At high loading levels (> 60%), the AE signals from the 90° laminated rocks exhibit a significant increase, highlighting reduced energy release during the pre-damage phase, whereas intense AE activities suggest that damage culminates in sudden failure at the damage threshold.

#### AE b-value analysis

The AE *b*-value serves as an indicator of the distribution of magnitude scale for AE events, reflecting the relative frequency of rupture occurrences within the rock. This metric enables the analysis and identification of precursors to rock rupture^34^. The characteristics associated with variations in the *b*-value hold significant interpretative value^32^. Specifically, an increase in the *b*-value suggests a higher relative occurrence of small-scale ruptures, whereas a decrease indicates a rise in the frequency of large-scale rupture events. Furthermore, minor fluctuations in the *b*-value may correspond to a process of gradual stabilization and expansion leading to rupture, while significant and abrupt changes in the *b*-value typically signal sudden destabilization and damage. The concept of the *b*-value was first introduced by Gutenberg and Richter^35^ in their investigations of seismic activity, where they observed that the logarithm of the cumulative number *N* of regional earthquakes exceeding a specific magnitude *M* decreases linearly with increasing magnitude *M*,

9 $$LgN=a - bM$$

where *a* and *b* are constants. In rock chamber tests, the magnitude is replaced by the amplitude^27^ for calculating the AE *b*-value:

10 $$Lg\;N = a - b(A_{{dB}} /20)$$

in which *A*_dB_ is the maximum amplitude of the AE signal in decibels (dB), and it is defined as *A*_dB_ = 20lg(*A*/*A*_0_), where *A* is the maximum amplitude of the AE signal and *A*_0_ is the reference amplitude.

**Fig. 9:**
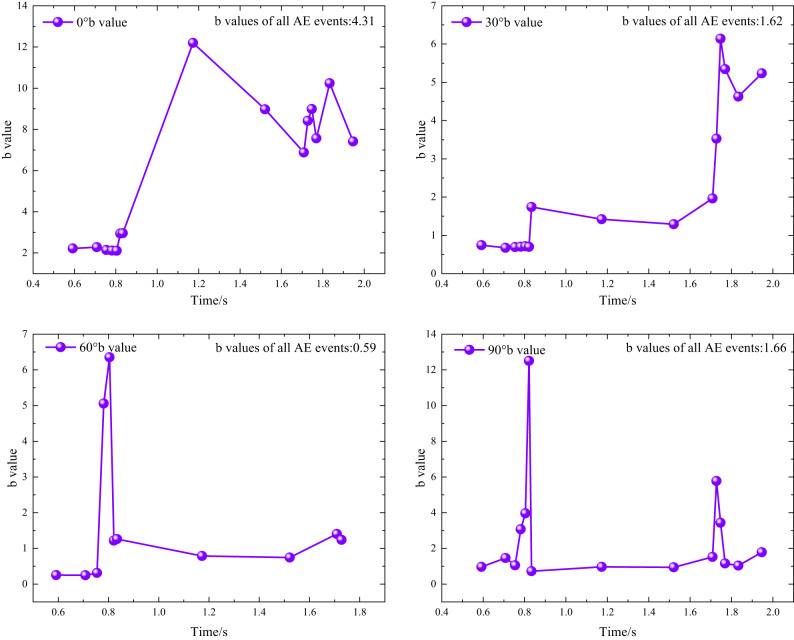
AE *b*-values of rocks with various lamination angles under impact loading.

The Fig. 9 illustrates the temporal evolution of the AE *b*-value for rocks at various lamination angles (0°, 30°, 60°, 90°) at the same impact pressure.

The *b*-value serve as a crucial parameter for characterizing damage progression and rupture in rocks, often utilized to analyze the development of microcracks and the trends associated with destabilizing damage. Given that the available data in impact tests are relatively limited compared with static tests, a sliding-window approach was adopted to compute the $\:b$-value. According to previous studies, an overlap of approximately 50% between adjacent windows generally yields better performance^32,36^. Considering the characteristics of the impact experiments as well as the selected window size and step length, a window of five AE events and a step of two AE events were used in this study to enhance computational stability and to reduce the influence of data dispersion on trend evaluation. Meanwhile, different window-parameter settings were compared, and the overall evolutionary trends were found to remain consistent.

To avoid confusion, the time axis in Fig. 9 corresponds to the entire AE recording window rather than the duration of the stress wave; therefore, the two are not of the same order of magnitude. Moreover, in impact tests, effects such as stress-wave reflections within the rock specimen can lead to an excessive number of near-threshold events, whereas large-amplitude hits are scarce, which tends to increase the computed *b*-value^34^. In addition, because impact loading occurs at a high rate, the amount of acquired data is limited. To analyze the entire impact process in detail, relatively small window sizes and step lengths are often adopted; however, this can make the fitted slope highly sensitive to slight deviations in the data distribution during acquisition, resulting in abnormally large *b*-values^37^. These factors only affect the numerical $\:b$-value results and do not alter the overall trend-based conclusions. If high-precision $\:b$-value data are required, AE instrumentation with a higher acquisition (sampling) frequency would be necessary.

The overall trend of the *b*-value indicates that the curves for all lamination angles exhibit nonlinear fluctuations, signifying that the processes of crack initiation, expansion, and destabilizing damage evolve dynamically throughout loading. With respect to the peak value of the instantaneous *b*-value. The *b*-values for 0° and 90° laminations show maximum peaks in the range of 12–14, whereas those for 30° and 60° are only between 6 and 7, reflecting a nearly twofold difference. This suggests that the *b*-values for the 0° and 90° laminations remain within the range of 12–14. When the lamination angle is misaligned with the loading direction, the presence of laminae reduces the destructive impact of stress waves on the rock; hence, loading in perpendicular or parallel orientations facilitates fracture and fragmentation.

All angles of rock laminations under impact loading have experienced significant sudden shifts in *b*-values, indicating that these rocks have undergone abrupt destabilizing damage. Notably, the *b*-values for both the 0° and 90° stratigraphy angles exhibit gradual changes within a narrow range, suggesting a progressive stabilization and expansion of rupture.

In terms of the $\:b$-value derived from the full-duration statistics of AE events. For the 0° stratigraphic angle, the AE *b*-value for all AE events is relatively high, at 4.31. This observation indicates that during loading, the propagation of fine cracks predominates, resulting in a complex and scattered small-scale fracture network within the rock body, reflecting a relatively smooth damage profile. In contrast, for the 30° stratigraphic angle, the AE *b*-value is lower at 1.62, indicating an increasing prevalence of larger cracks as the stratigraphic angle rises. Here, small-scale crack development is gradually overtaken by large-scale cracks. At the 60° angle, the AE *b*-value drops to a minimum of 0.59, considerably lower than that of the other stratigraphy angles, indicating that the rock is primarily characterized by the proliferation of a few large-scale cracks, indicative of pronounced centralized brittle damage. The *b*-value at the 90° angle, recorded at 1.66, lies between those of the 30° and 60° angles, suggesting an intermediary state of crack development, with a notable presence of large-scale cracks that have not reached the extreme concentration observed at the 60° angle.

From the perspective of damage–fracture evolution, the $\:b$-value can be used to characterize the transition from “diffuse microcrack growth” to “crack localization and main-crack formation”. A higher $\:b$-value implies that AE activity is dominated by small-scale fracture events, corresponding to multi-site crack nucleation, dispersed energy dissipation, and the absence of a dominant macroscopic crack pathway. A sustained decrease in the $\:b$-value, by contrast, reflects coalescence and localization of cracking at larger scales, with the main crack progressively becoming dominant and the system entering a stage closer to unstable propagation. In particular, an abrupt drop or jump in the $\:b$-value often indicates a sudden increase in the proportion of large-scale events and accelerated structural instability, which is consistent with the manifestation of “sudden unstable failure”.

From an engineering standpoint, the long-term trend and abrupt changes of the $\:b$-value can serve as indicators for assessing instability risk of laminated rock masses under impact disturbances: (1) when the $\:b$-value remains high with only minor fluctuations, it suggests that damage is mainly governed by dispersed microcrack activity and the failure process is relatively progressive; (2) when the $\:b$-value decreases continuously or exhibits a pronounced drop within a short time window, it implies an increasing prevalence of large-scale cracking events and rapid formation of a main crack, making the structure more likely to undergo localized brittle failure.

### Crushing characteristics of laminated rocks under impact loading

The mass-equivalent size method was employed to determine the fractal dimensions of rock fragmentation, as detailed in^38^. This involved conducting a sieve test using a series of 9 standard sieve grades ranging from 2.36 mm to 37.5 mm. The mass of the rock fragments retained on each sieve was measured and subsequently converted into the cumulative percentage of the under-sieve content for each sieve size. Utilizing the constructed mass-frequency function of the fragmentation distribution, alongside the sieve data, enabled the derivation of the fractal distribution of the rock specimens tested in Hopkinson bar process, thereby facilitating the development of a formula for fragmentation distribution:

11 $$M(L)/M={\left( {L/{L_{\mathrm{m}}}} \right)^{3 - D}}$$

where *D* represents the fractal dimension of impact-crushed rock; *L* denotes the size of the sieve opening; *M* indicates the mass of the specimen prior to crushing; *L*_*m*_ refers to the grain size of the specimen following the crushing process; and *M*(*L*) signifies the mass retained by the sieve. By applying a logarithmic transformation to both sides of the equation, one can arrive at the following relation:

12 $$\lg [M(L)/M]=(3 - D)\lg \left( {L/{L_{\mathrm{m}}}} \right)$$

The curve fitting was performed using lg(*L*/*L*_*m*_) as the horizontal coordinate and lg(*M*(*L*)/*M*) as the vertical coordinate.

**Fig. 10:**
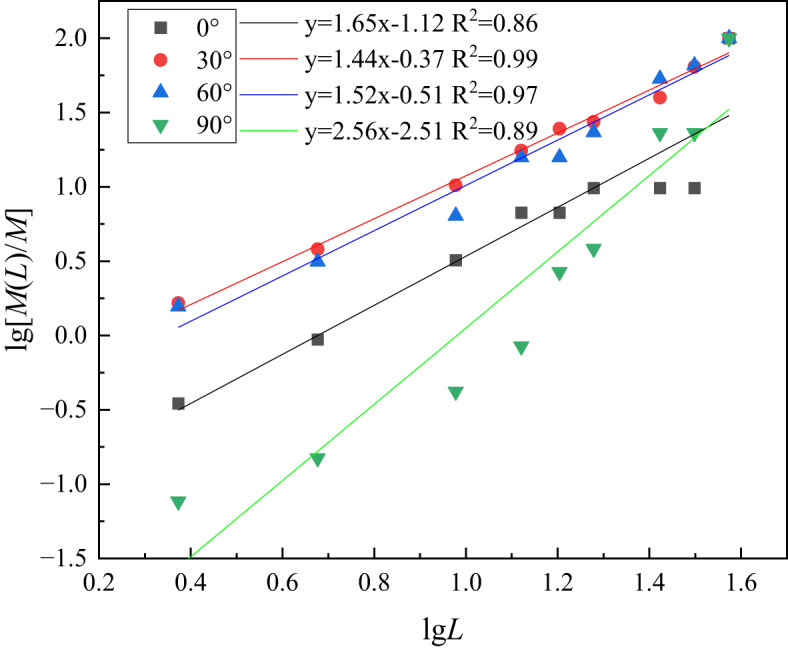
Fitting of sieving data for laminated rocks.

The slope obtained from the fit in Fig. 10 is 3-*D*, which leads to the fractal dimension *D*.

**Fig. 11:**
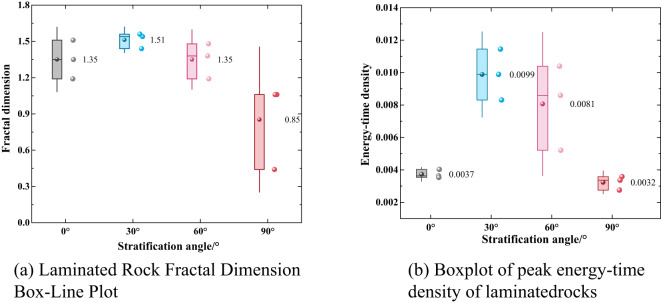
Plots of the fractal dimension and the peak energy-time density data for various laminated rocks. (**a**) Laminated Rock Fractal Dimension Box-Line Plot, (**b**) Boxplot of peak energy-time density of laminated rocks.

The fractal dimension is commonly regarded as an effective indicator of the degree of rock fragmentation: a higher fractal dimension implies more thorough fragmentation. The energy–time density represents the intensity of energy dissipation per unit rock volume per unit time. In general, a larger energy–time density indicates stronger and more sufficient energy dissipation, and thus a higher degree of rock fragmentation^39,40^. As shown in Fig. 11, the energy–time density exhibits a certain correlation with the fractal dimension of rock fragmentation; however, given the relatively small dataset, further in-depth investigations are required to quantify the strength of this correlation. In addition, comparisons across different bedding angles indicate that the angle between the bedding orientation and the impact direction significantly affects the fragmentation degree: when the impact direction is perpendicular or parallel to the bedding, both the energy–time density and the fractal dimension tend to be lower; whereas when the impact direction forms an oblique angle with the bedding, the energy–time density and fractal dimension are higher, which is more conducive to enhanced rock fragmentation.

## Concluding remarks

In this study, systematic impact tests were conducted on laminated rocks under a constant gas pressure. By introducing an energy–time density index and integrating digital image correlation (DIC) and acoustic emission (AE) monitoring, together with sieving analyses of the resulting fragments, the energy dissipation, fracture modes, and fragmentation characteristics of laminated rocks under impact loading were systematically investigated. The main conclusions are as follows:

1. The bedding angle exerts a significant influence on the evolution of the rock energy–time density. When the bedding orientation is oblique to the loading direction, the intensity of energy dissipation is higher, accompanied by more complex shear–tensile mixed failure and faster propagation of large-scale cracks. In contrast, when the bedding orientation is parallel or perpendicular to the stress-wave propagation direction, the energy-dissipation intensity is lower and the failure mode becomes more single and simplified.
2. At low loading levels, the rock mainly undergoes elastic deformation with limited crack formation. At intermediate loading levels, cracks propagate actively and are accompanied by pronounced shear damage. At high loading levels, AE activity increases markedly prior to critical failure, exhibiting a sudden-failure characteristic. The *b*-value analysis indicates that the 0° laminated rock shows a relatively high *b*-value, implying the dominance of small-scale cracking and a more stable crack-evolution process; in contrast, the 60° laminated rock is characterized by rapid growth of small-scale cracks that ultimately develop into large-scale failure, suggesting faster structural destabilization and more violent damage.
3. There is a correlation between energy–time density and fractal dimension, indicating that improved fragmentation efficiency can be achieved under specific bedding orientations. This finding not only deepens the understanding of the response mechanisms of anisotropic rocks, but also provides important guidance for safety management of critical infrastructure, including mining, tunneling, and underground engineering, thereby enabling more accurate prediction and mitigation of impact-induced failure.
4. Owing to limitations associated with a single lithology, uniform loading conditions, and the experimental equipment, the results of this study mainly reflect general trends. Future work may be extended to a wider range of lithologies, more complex strain-rate conditions, and more precise quantitative outcomes, with tailored experimental designs as needed to reduce the risk of structural instability.

## Data Availability

The data presented in this study are available on request from the corresponding author due to institutional confidentiality agreements and data privacy considerations.
